# Urethral catheterization as an alternative method for collecting sperm in the black-footed ferret (*Mustela nigripes*)

**DOI:** 10.1093/conphys/coaa078

**Published:** 2020-08-25

**Authors:** Paula Mackie, Biankha Chan, Maria Franke, Gabriela F Mastromonaco

**Affiliations:** Wildlife and Science Division, Toronto Zoo, Scarborough, ON M1B 5K7, Canada

**Keywords:** Assisted reproductive technologies, electroejaculation, ferret, semen analysis, urethral catheterization

## Abstract

The endangered black-footed ferret (BFF; *Mustela nigripes*) is an important example of the benefits of assisted reproduction in species conservation with both semen evaluation and artificial insemination using fresh and frozen sperm being successfully incorporated into the breeding program. Currently, electroejaculation (EE) is routinely utilized for semen collection in BFFs, a technique that requires custom equipment and experienced operators, and does not consistently yield viable samples in this species. In this case study, we evaluated the feasibility of urethral catheterization (UC) for semen collection, a method predominately tested in domestic and non-domestic felids, on four occasions (three BFF males). After general anesthesia with a combination of ketamine, midazolam and α2-agonist dexmedetomidine (thought to promote semen release into the urethra), a lightly lubricated, flexible feeding tube was passed into the urethral opening and advanced ~7–8 cm into the urethra. A syringe attached to the feeding tube was used to apply mild negative pressure to collect sperm. Semen samples were successfully collected on all four attempts. Sperm characteristics ranged as follows: 10.5–26.0 × 10^6^ sperm/ml concentration, 50–90% motility and 36–61% normal sperm morphology. This is the first report of the use of UC as a potential alternative to EE in the BFF, a more field-friendly technique that is less invasive and more consistent for obtaining samples free of urine contamination.

## Introduction

Assisted reproductive technologies (ARTs), particularly artificial insemination (AI), have the potential to significantly enhance the genetic management and long-term conservation of threatened wildlife species. ARTs can be used to improve the reproductive performance of captive individuals, ensure the contribution of genetically distinct individuals and mitigate behavioural incompatibilities or other health-related issues (Howard and Wildt, 2009). Furthermore, cryopreserved sperm from genetically valuable males can be stored in biobanks for several generations providing insurance against a sudden or drastic loss of genetic diversity and avoiding genetic drift in small populations (Wildt and Roth, 1997; Wildt and Wemmer, 1999).

Assisted reproduction has played an important role in the current success of the black-footed ferret (BFF; *Mustela nigripes*) recovery strategy. In 1988, a recovery program was initiated with the remaining 18 BFFs, which emphasized a multi-institutional *ex situ* breeding program and the development of ARTs for the species (US Fish and Wildlife Service, 1988). From seven founder individuals, the captive breeding population is currently > 300 animals across six institutions, while more than 4300 ferrets have been released back into the wild since 1991 (Santymire and Graves, 2019). More importantly, AI was involved in the production of ~ 140 offspring using fresh and frozen/thawed semen (Howard and Wildt, 2009).

The collection of semen is a fundamental step for fertility assessment, assisted reproduction and genome banking (Lueders *et al*., 2012). Electroejaculation (EE) has been employed as the method of choice for most non-domestic species due to its ability to collect semen from males that are unable to mate naturally due to physical or behavioural handicaps (Leibo and Songsasen, 2002; Tecirlioglu *et al*., 2002). EE uses rectal probes that release increasing electrical pulses in a stepwise fashion until ejaculation is achieved (Brindley, 1981). However, EE requires expensive and custom equipment and an experienced operator; furthermore, permission to use the technique is not readily granted in many parts of the world, including European countries (Falk *et al*., 2001; Zambelli *et al*., 2008). Lastly, utilization of EE for *in situ* sperm collection is complicated by the need for electricity that can prove impractical in remote locations.

Urethral catheterization (UC) after medetomidine administration, also known as the ‘Zambelli’ method, is a novel technique that has been successfully performed in domestic cats (*Felis catus—*Zambelli *et al*., 2008), African lions (*Panthera leo*—Lueders *et al*., 2012), Asiatic golden cats (*Catopuma temminckii*—Lueders *et al*., 2014), jungle cats (*Felis chaus*—Kheirkhah *et al*., 2017), Amur leopards (*Panthera pardus orientalis*—Jeong *et al*., 2018), polar bears (*Ursus maritimus*—Curry and Roth, 2015) and Asiatic black bears (*Ursus thibetanus*—Jeong *et al*., 2019). Zambelli *et al*. (2008) found that sperm concentration following EE was significantly higher after the administration of medetomidine compared to ketamine. The ductus deferens is thought to contain alpha-adrenoreceptors, and therefore, alpha-2 agonists, such as medetomidine, may enhance smooth muscle contractions of the ductus deferens causing semen to be released into the pelvic urethra (Turner *et al*., 1995; Johnston and Deluca, 1998). UC has been found to result in lower semen volume, but higher sperm concentration and lower urine contamination in comparison to EE (Zambelli *et al*., 2008; Jeong *et al*., 2019). Higher-quality semen samples, minimal invasiveness and lower cost make UC an attractive alternative toEE.

Currently, EE is routinely used for semen collection in BFFs with variable success. The purpose of this case study was to evaluate the feasibility of the ‘Zambelli’ method for reproductive assessment of male BFFs prior to breeding introductions in captivity or for future use in thewild.

## Materials and methods

### Animals and housing

All protocols and procedures were conducted in accordance with the Toronto Zoo Animal Care and Research Committee guidelines for animal use. Three male BFFs aged 10 months to 4 years were included in the study over two consecutive breeding seasons (2018 and 2019; one animal repeated in both years). Animals were housed indoors at an ambient temperature of 19–22 °C in individual pens (84 cm × 213 cm × 92 cm) illuminated by overhead fluorescence bulbs on a changing light:dark cycle to mimic natural seasonal lighting conditions (US Fish and Wildlife Black-footed Ferret Managed Care Operations Manual). Males were fed 60–70 g of Toronto Zoo Small Carnivore Diet (Milliken Meat Products; Scarborough, ON, Canada) daily, supplemented with a whole animal (mouse or rat) twice weekly. Testicular firmness was manually assessed daily and graded on a scale of 1–3 (1-firm, 3-flaccid). Semen collection was only attempted on animals with a sustained testicular firmness of 1–1.5 for at least 30 days.

### Anesthesia and semen collection

Animals were anesthetized using an intramuscular (i.m.) combination of ketamine (Ketaset®; 2 mg/kg; Zoetis Canada Inc. Kirkland, QC, Canada), midazolam (Midazolam sterile injectable solution; 0.2 mg/kg; Sandoz Canada Inc. Boucherville, QC, Canada) and dexmedetomidine (Dexdomitor®; 25 ug/kg; Orion Pharma, Espoo, Finland). Additional ketamine was administered subcutaneously (s.c) throughout the procedure as required. Based on a previous study in cats, semen collection by UC was conducted no sooner than 25 minutes post anesthesia induction to ensure adequate time for the dexmedetomidine to stimulate semen release into the ureter (Swanson *et al*., 2016).

**Figure 1:**
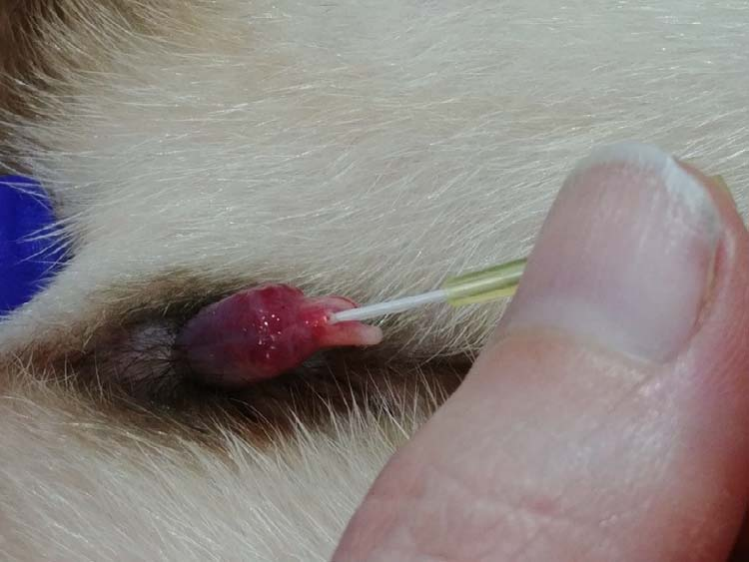
Location and distension of the opening of the urethra using an intravenous catheter without the needle and the groove of the baculum as a guide

**Figure 2:**
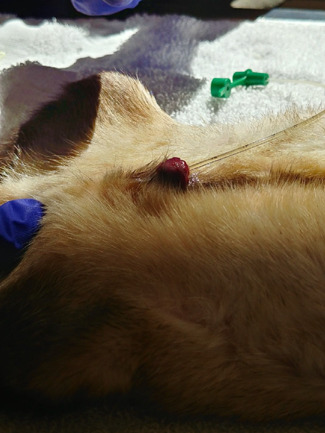
Placement of the feeding tube in the urethra

Following Brown and Pollock (2010), the location and distension of the urethral opening was achieved by the insertion of a 24 G intravenous catheter without the needle, using the groove of the baculum as a guide (Fig. 1). The opening is ~4 mm from the distal tip of the J-shaped penis. A lightly lubricated (Priority Care, First Priority, Inc., IL, USA; non-spermicidal lubricant) 3.5 Fr × 31 cm flexible argyle feeding tube (cat# 8888-261206, Sherwood Medical, St. Louis, Missouri, USA or cat#461206, Covidien Ilc, Mansfield, MA, USA) was inserted adjacent to the intravenous catheter. Following successful insertion, the intravenous catheter was slowly removed and the feeding tube was advanced ~7-8 cm into the urethra (Fig. 2). Negative pressure was created by applying a mild suction (0.1–0.2 ml) using a 3 ml syringe attached to the end of the catheter. After 30 seconds *in situ*, the catheter was retracted 4 cm, and 0.2 ml of negative pressure was applied and held for an additional 30 seconds before retraction of the catheter from the penis. Urine contamination in the catheter was noted. Semen was immediately ejected into a 1.5 ml microcentrifuge tube containing 200 μl of pre-warmed (37 °C) semen extender (TEST yolk refrigeration medium; Irvine Scientific, Santa Ana, CA, USA) and held at 37 C.

### Semen evaluation

Samples were microscopically assessed for the presence or absence of spermatozoa, and subsequently evaluated for motility (subjectively; 0–100%), progressive motility (0–5 scale; 0 = non-motile and 5 = rapid forward movement) and concentration using a hemocytometer. Due to the minimal volumes (<10 μl) of semen retrieved from the catheter, sperm concentration and total sperm count were calculated based on the initial 200 μl dilution volume of extender. A small volume was also smeared on a microscope slide, which was then fixed in 1% paraformaldehyde before staining (Spermac Stain; FertiPro NV, Beernem, Belgium) to evaluate sperm morphology.

## Results

**Figure 3:**
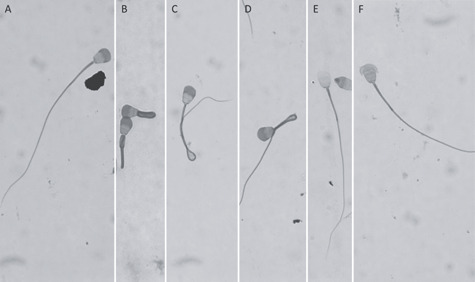
Morphological assessment of sperm stained with Spermac® showing normal and predominant abnormalities observed. (**A**) normal, (**B**) coiled tail, (**C**) bent tail, (**D**) bent mid-piece, (**E**) absent acrosome, (**F**) damaged acrosome.

Semen was successfully collected from all three males (four attempts, one male repeated in each collection year). During the procedure, resistance occurred 6 cm post insertion (tip of catheter palpable at the caudal flexion of the penis, immediately caudal to the proximal baculum) but could be easily passed with gentle manipulation. In male ‘1’, a different catheter (Covidien) was attempted and proved to be more difficult to insert, resulting in a delay of 70 minutes from injection of anesthetic to collection. Sperm motility ranged from 50% to 90% with progressive motility ranging from 3–5 (Table 1). Sperm concentration ranged from 10.5–26.0 × 10^6^/ml (Table 1). Normal sperm morphology ranged from 36–61% with coiled tails, bent tails and mid-pieces and damaged or absent acrosomes being the predominant abnormalities (Table 1, Fig. 3). Urine contamination was not observed in any of the samples.

**Table 1:** Evaluation of sperm characteristics following collection byUC

	Individual			
	1	2 (2018)	2 (2019)	3
Age (year)	4	1	2	2
Time from injection to collection (min)	81	30	27	35
Motility (%)	90	60	90	50
Progressive motility (0–5)	4–5	2	4	3–4
Sperm concentration (×10^6^/ml)^a^	15	10.5	26	18.5
Total sperm count (×10^6^)^a^	3	2.1	5.2	3.7
Normal morphology (%)	59	49	61	36
Abnormal morphology^b^:				
Coiled tails (%)	5	23	12	26
Bent tail (%)	7	11	16	17
Bent midpiece (%)	2	2	4	8
Damaged acrosome (%)	32	NR	33	32
Absent acrosome (%)	27	NR	10	18

a ^a^ Calculation based on initial volume of extender present in collectiontube.

b ^b^ Only commonly observed abnormalities are listed. NR = data not recorded.

## Discussion

To our knowledge, this is the first study to demonstrate the use of UC to successfully obtain semen from BFFs on all four attempts. Currently in BFFs, semen collection is routinely carried out using EE, a protocol that can only be undertaken by facilities having the necessary equipment and trained operators. Furthermore, the technique has been difficult to implement consistently and successfully in this species. Our results indicate that UC can be considered as an alternative method for semen collection and reproductive assessment in BBFs. UC has been described as being less invasive, less expensive and quicker to perform than EE, as well as producing samples with lower urine contamination and higher sperm concentration compared to EE (Zambelli *et al*., 2008; Lueders *et al*., 2012; Swanson *et al*., 2016; Jeong *et al*., 2018). Additionally, unlike EE, UC does not require electricity, which, combined with the ease of implementation, makes this a more feasible technique for *in situ* collections. Notably, utilization of medetomidine in the induction cocktail, which is not routinely used with ferrets in our facility, did not have any adverse effects on the animals or the procedure.

Studies collecting semen via EE in BFFs reported an average volume of 26.9 ± 3.1 μl and sperm concentration of 558.6 ± 121.5 × 10^6^/ml (Santymire *et al*., 2006). Howard *et al*. (2015) documented similar values with average ejaculate volume of 20 ± 0.2 μl and sperm concentration of 772.7 ± 133.3 × 10^6^/ml. In the current study, samples were immediately diluted into sperm extender (TEST) to conserve sperm viability, preventing accurate volume measurements. Although the UC method resulted in lower semen concentration in comparison to previous reports using EE, these outcomes may have been influenced by training period (i.e. implementation of a new technique), low sample size and individual reproductive status of the males. Furthermore, catheterization was not repeated if an adequate number of sperm were observed, possibly resulting in incomplete collection of all available sperm in the urethra. Reports describing sperm morphology and incidence of abnormalities vary greatly in this species, and appear to be impacted by age (Wolf *et al*., 2000) and level of inbreeding (Santymire *et al*., 2018). Sperm morphology following UC was similar to that described previously (0–89%, μ = 33 ± 1.1; Santymire *et al*., 2018) with the predominant abnormalities including damaged acrosomes, coiled tails and bent tails or midpieces.

Unexpectedly, in this small sample size, the type of catheter greatly impacted the ease of insertion into the urethra. Although both catheters, Sherwood Medical and Covidien, were made of polyvinylchloride, identical in size (3.5 Fr) and lumen opening shape, and labelled as feeding tubes, the Sherwood Medical feeding tube was much easier to insert and advance into the urethra allowing for more rapid sperm collection (<5 minutes) compared to the Covidien feeding tube (>1 hour). We were also unable to discern a palpable difference between the catheters, but we suspect a slight variance in rigidity, despite reassurance from the manufacturer that there is no difference in their composition. Clinically, recommendations for urethral catheters in ferrets to clear urinary blockages include opaque 3.0 Fr silicone catheters known as ‘Slippery Sam’, 3.5 Fr red rubber catheters or 3.5 Fr Tom cat catheters (Marini *et al*., 1994; Brown and Pollock, 2010; Hoefer, 2013). In our experience, clear flexible tubing is ideal for easy manipulation and visualization allowing the operator to determine a successful collection without needing to fully remove the catheter before repositioning.

Our attempts to perform EE in BFFs have been met with limited success with negligible volume and sperm being obtained during most retrievals. Although the sperm concentration reported in this case study was lower, the samples obtained following UC were a significant improvement to previous attempts with EE. This suggests that further investigation of UC is warranted in this and other small, difficult-to-collect species. Most importantly, development of this minimally invasive and easily applicable technique could be a valuable alternative for researchers working with small mammals in the field.

## Funding

This work was supported by the Toronto Zoo.
